# Anticancer agent α-sulfoquinovosyl-acylpropanediol enhances the radiosensitivity of human malignant mesothelioma in nude mouse models

**DOI:** 10.1093/jrr/rrab090

**Published:** 2021-11-05

**Authors:** Eiko Inamasu, Tomoshi Tsuchiya, Motohiro Yamauchi, Kodai Nishi, Katsuya Matsuda, Fumio Sugawara, Kengo Sakaguchi, Ryoichi Mori, Keitaro Matsumoto, Takuro Miyazaki, Go Hatachi, Ryoichiro Doi, Hironosuke Watanabe, Koichi Tomoshige, Naoki Matsuda, Yoshikazu Higami, Isao Shimokawa, Masahiro Nakashima, Takeshi Nagayasu

**Keywords:** malignant pleural mesothelioma (MPM), radiosensitizer, hypoxia-inducible factor (HIF), sulfoquinovosyl-acylpropanediol (SQAP)

## Abstract

Malignant pleural mesothelioma (MPM) is a highly malignant disease that develops after asbestos exposure. Although the number of MPM cases is predicted to increase, no effective standard therapies have been established. The novel radiosensitizer α-sulfoquinovosyl-acylpropanediol (SQAP) enhances the effects of γ-radiation in human lung and prostate cancer cell lines and in animal models. In this study, we explored the radiosensitizing effect of SQAP and its mechanisms in MPM. The human MPM cell lines MSTO-211H and MESO-4 were implanted subcutaneously into the backs and thoracic cavities of immunodeficient KSN/Slc mice, then 2 mg/kg SQAP was intravenously administered with or without irradiation with a total body dose of 8 Gy. In both the orthotopic and ectopic xenograft murine models, the combination of irradiation plus SQAP delayed the implanted human MSTO-211H tumor growth. The analysis of the changes in the relative tumor volume of the MSTO-211H indicated a statistically significant difference after 8 Gy total body combined with 2 mg/kg SQAP, compared to both the untreated control (*P* = 0.0127) and the radiation treatment alone (*P* = 0.0171). After the treatment in each case, immunostaining of the harvested tumors revealed decreased cell proliferation, increased apoptosis and normalization of tumor blood vessels in the SQAP- and irradiation-treated group. Furthermore, hypoxia-inducible factor (HIF) 1 mRNA and protein expression were decreased, indicating reoxygenation in this group. In conclusion, SQAP improved hypoxic conditions in tumor tissue and may elicit a radiosensitizing effect in malignant mesothelioma models.

## INTRODUCTION

Malignant pleural mesothelioma (MPM) is a refractory malignancy that arises from mesothelial cells covering the thoracic and abdominal cavity and is mainly caused by asbestos deposition [1,2]. Epidemiologically, the number of patients will gradually increase and is expected to peak around 2020 globally and around 2030 in Japan [3]. The prognosis is extremely poor with half of all patients dying within two years. Although trimodal treatment with chemotherapy (cisplatin plus pemetrexed), surgery (total pleuropulmonary resection or pleural decortication) and radiation therapy (intensity modulation radiation therapy [IMRT]) is performed as a treatment strategy [4], it causes great damage to the body, thus compromising its therapeutic effect. Because the radiation field reaches half of the thoracic cavity, controlled field reduction, which is limited to the chest wall and excludes the lungs, has been attempted with IMRT. However, IMRT requires a 50–60 Gy total body dose irradiation level and the high level and limited irradiation often causes radiation pneumonitis and radiation-related esophagitis [5,6]. Therefore, the radiation dose should be reduced to ensure therapeutic efficacy.

The radiosensitizer α-sulfoquinovosyl-acylpropanediol (SQAP) was developed based on the natural product, sulfoquinovosyl acylglycerol, found in seaweed cedar, which is a glycolipid with almost no cytotoxicity but has a strong radiosensitization rate (≥3.0) [7–15]. To date, several radiosensitizers have been reported including gemcitabine and pentoxifylline [16–18]. *In vivo*, gemcitabine was shown to have a dose modifying factor (DMF) of 1.1–2.4. In clinical use, however, there are many adverse events, and concomitant use of gemcitabine and RT currently cannot be recommended [17]. Pentoxifylline and the related methylxanthine caffeine enhance radiation effects. However, these effects are cell line-dependent and require mutant or dysfunctional p53 [19]. Therefore, radiation sensitizers that have shown promise have yet to be realized.

SQAP accumulates in malignant cells, temporarily reoxygenates the solid cancer tissue immediately after administration, and synergistically increases the radiation effects [20]. *In vivo* mouse models of lung and prostate cancer have shown that the likely molecular mechanisms include tumor oxygenation by facilitating oxygen dissociation from oxyhemoglobin and inhibiting the hypoxia-inducible factor (HIF) pathway. [14,20]. Therefore, we hypothesized that SQAP may have similar antitumor effects in MPM. In this study, we demonstrated the additive effect of SQAP as a radiosensitizer in ectopic (skin) and orthotopic (thoracic cavity) malignant mesothelioma mouse models and revealed the probable mechanisms of the effect.

## MATERIALS AND METHODS

### Cell lines and cultures

Human MPM cell lines MSTO-211H and MESO-4 were purchased from the American Type Culture Collection (Manassas, VA, USA). The cells were maintained in RPMI 1640 (Thermo Fisher Scientific K.K., Yokohama, Japan) supplemented with 10% heat-inactivated fetal bovine serum (Gibco, Grand Island, NY, USA), penicillin (100 U/ml), and streptomycin (50 μg/ml). All cell lines were incubated at 37°C in a humidified atmosphere with 5% CO_2_. We consistently used 90% confluent cells during this study.

### Drugs

**Fig. 1:**
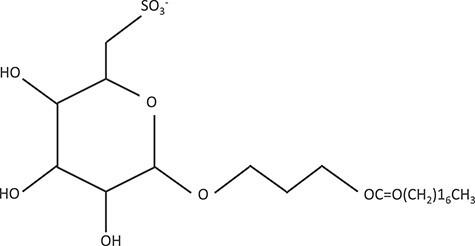
Molecular structure of SQAP. SQAP is found in seaweed cedar and is a glycolipid with almost no cytotoxicity. SQAP, α-sulfoquinovosyl-acylpropanediol.

SQAP (Fig. 1) is a glycolipid consisting of a molecule of glucose and a molecule of stearic acid with a sulfone group at position 6 connected by a propanediol. The SQAP was synthetized as an analogue of Sulfoquinovosyl-acylglycerols (SQAG), originally isolated from natural sources such as higher plants, sea urchins and marine algae. SQAP was provided by CANGO (Tokyo, Japan) and dissolved in saline. Since approximately 2 mg/kg was used per mouse, the dose was adjusted to 0.6 mg/ml. The dissolved solution was stored at 15°C and was used within a day. Quality control was performed using NARD CHEMICALS, LTD. The synthesized material number (I-01; 2.8 kg) was analyzed based on appearance, underwent confirmation testing using the IR-KBr tablet method and purification testing, and calcium content and SQAP content were assessed using the titration method. The sample was a white powder. The IR-KBr tablet method proved that the content was attached to the reference spectrum. The analogues were found to be <1%. Residual levels of methanol, toluene and methylethylketone were lower than 99, 101 and 50 ppm, respectively. The calcium content was 3%. SQAP content was 98.1%.

### Radiation dosimetry and treatment

Irradiation was performed at room temperature in a ^137^Cs γ-ray irradiator at a dose rate of 1 Gy/min measured with a NIST-based RAMETEC1000plus radiation (TOYO MEDIC CO., LTD., Tokyo, Japan) equipped with Model N23323 intracavitary ion chamber.

**Fig. 2:**
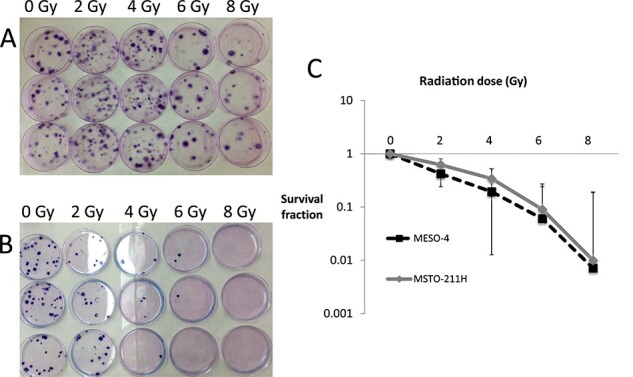
Radiosensitivity of two mesothelioma cell lines. Colony forming assays of MSTO-211H biphasic-type mesothelioma cells (A) and MESO-4 epithelioid-type mesothelioma cells (B). Dose-survival curves of MSTO-211H and MESO-4 cells after irradiation (C). No significant differences in radiosensitivity are shown but MESO-4 cells showed slightly higher radiosensitivity even at a lower irradiation doses.

### Colony formation assays

Clonogenic cell survival was evaluated using dose-survival curves and colony formation assays. Tumor cells that had not been previously irradiated were irradiated in plastic tubes, then seeded in 100-mm dishes. The 100-mm dishes with 100 cells and 10 ml medium were placed directly on the turntable of the irradiator and received one irradiation of 2, 4, 6 or 8 Gy, and incubated at 37°C for 10–14 days. The plating efficiency (PE) was calculated as the number of colonies divided by the number of seeded cells in the untreated control. The measured surviving fraction of the irradiated samples (the number of colonies/the number of seeded cells) is then normalizes to the PE of the unirradiated controls. We repeated the study three times and confirmed its reproducibility. The scoring was evaluated by two blinded researchers.

### Establishment of *in vivo* ectopic and orthotopic xenograft models and treatments

Five- to eight-week-old male nude mice (KSN/Slc) were purchased from Japan SLC, Inc. (Fukuoka, Japan) and housed under specific-pathogen-free conditions. Mice were housed in cages with no more than three mice per cage. The Institutional Animal Care and Use Committee (IACUC) approved all animal experiments (study number 1510191255), which were performed in compliance with the Animal Welfare Act. All surgery was performed under sodium pentobarbital anesthesia, and all efforts were made to minimize suffering.

Mice were immediately euthanized by CO_2_ inhalation following an Institutional Animal Care and Use Committee approved protocol in which human study endpoint criteria were observed. Study endpoint criteria included obvious distress, failure to ambulate and weight loss >20%. Animal health, behavior and body weight were monitored once a day.

MSTO-211H and MESO-4 cells (5 × 10^6^ cells/mouse) suspended in phosphate-buffered saline were injected subcutaneously or into the pleural cavity. *In vivo* orthotopic MPM xenograft models were established as described previously [7]. After adequate anesthesia, 5.0x10^6^ MSTO-211H or MESO-4 cells suspended in 2 ml of phosphate-buffered saline were injected either subcutaneously for ectopic, or in the right pleural cavities of five- to eight-week-old male KSN mice. When tumor cells were accidently injected into incorrect body locations, or showed evidence of pneumothorax or hemothorax, the animals were euthanized via excess anesthesia and excluded from further analyses.

**Fig. 3:**
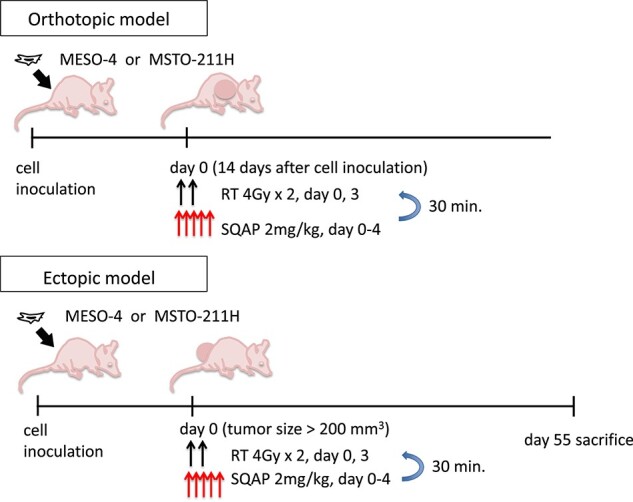
Timelines of experimental design of *in vivo* studies.

**Fig. 4:**
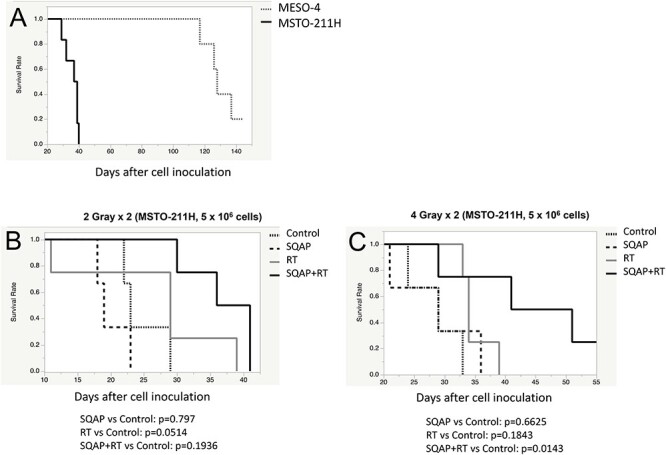
Establishment of orthotopic models of MPM and the therapeutic potential of SQAP. Survival curves of orthotopic models of two independent malignant pleural mesotheliomas (MESO-4; n = 5, MSTO-211H; n = 5)(A). The therapeutic effects of five injections of SQAP (2 mg/kg, i.v.) with or without two fractions of irradiation (B, C) 2Gray x 2 (B) or 4 Gray x 2 (C)/ fraction of irradiation were exposed to the MSTO-211H implanted mice (n = 3 or 4 for each group). Panel C shows a statistically significant difference of p = 0.0143 for SQAP+RT vs Control, but not for the other comparisons.

In the ectopic model mice, the tumor volume (*V* = mm^3^) and body weight were measured every 2 days and estimated using the equation, V = 0.5 × length × width^2^. When the estimated tumor volume reached >200 mm^3^, the mice were intravenously injected with either saline (control group) or SQAP (2 mg/kg, days 0–4) via tail vein. The injections to mice in the treatment groups receiving injections of saline or SQAP with radiation were completed 30 minutes prior to irradiation. Non-anesthetized mice were irradiated (4 or 2 Gy/fraction) on days 0 and 3 or days 0–4 with a γ-ray therapeutic machine (PS-3100SB; Industry Co., Ltd., Tokyo, Japan). Mice were placed in a cylindrical mouse holder and irradiated vertically to the dorsal side from a ^137^Cs source at a dose rate of 0.9Gy/min. Radiation dose was measured with a RAMETEC1000plus radiation system tied to a NIST-based dose gold standard. The variation of dose rate in the irradiation field was less than 5%. For MSTO-211H analysis, we analyzed six, eight, nine and seven mice in each treatment group (control, SQAP, RT and SQAP + RT, respectively). For the MESO-4 analysis, we analyzed four mice in each treatment group (control, SQAP, RT and SQAP + RT). The allocation was completely random, with individual differences in body weight at the start of the experiment being less than 5 g. Because some of the tumor-bearing mice could not eat by day 55, all mice were euthanized, and samples were harvested on day 55.

The harvested tumor samples were promptly divided into two at the point with maximum surface area. Half of the samples were fixed with 10% formalin, and the other half was further divided into two pieces. One was promptly stored at −80°C, and the rest was subjected to RNA degradation inhibition treatment (RNA later solution, Sigma-Aldrich, Tokyo, Japan) and stored at −80°C.

### Immunohistochemistry

Immunohistochemical staining was performed for all samples of ectopic models using Ki-67 and TdT-mediated dUTP nick-end labeling (TUNEL). For double immunofluorescence of CD31 and α-smooth muscle actin (α-SMA), sections from all samples were incubated at 4°C overnight with a rabbit anti-human CD31 antibody (1:200; Abcam, Cambridge, UK) and goat anti-human α-SMA antibody (1:200; Abcam), then with fluorescein isothiocyanate-conjugated anti-rabbit IgG (1:200; Invitrogen, Carlsbad, CA, USA) and Alexa Fluor 568-labeled anti-goat IgG (1:200; Invitrogen) for 1 h. Nuclei were counterstained with TO-PRO-3 iodine (1:1000, Invitrogen). Imaging was performed in a six-border zone region for each sample (All-in-one Fluorescence Microscope BZ-X700; Keyence, Osaka, Japan) and analyzed with a BZ-X Analyzer BZ-H3A. The number of analyzed samples was six, eight, nine and seven for the control, SQAP, RT and SQAP + RT groups, respectively. We observed 10 visual fields at 100× magnification and counted the number of positive cells in the evaluation of all immunostaining.

### Droplet digital PCR analysis

Tumor RNA was extracted from the tumor tissues of the MPM ectopic models using a QIA cube (All prep DNA/RNA mini kit; QIAGEN, Hilden, Germany). Digital droplet PCR was performed using a Droplet Digital PCR XQ200 system (Bio-Rad Laboratories, Hercules, CA, USA). PCR amplification in droplets was confirmed using the QX200 system (Bio-Rad). All experiments ware performed in triplicate. Several samples were excluded because of amount shortage. So the numbers of analyzed samples was 6, 7, 9 and 7 for the group of control, SQAP, RT and SQAP + RT, respectively.

### Statistical analysis

All data are expressed as the mean ± standard deviation. Differences between groups were examined for statistical significance using the Mann–Whitney U test and nonparametric analysis of variance. *P*-values of <0.05 were considered significant. For survival analysis, we used the Kaplan–Meier method and log-rank test.

## RESULTS

### Radiosensitivity of MSTO-211H and MESO-4 cells

Before examining the effects of SQAP in the animal models, we performed *in vitro* dose response studies using colony formation assays to determine the radiosensitivity of epithelial-type MPM MESO-4 cells and biphasic-type MPM MSTO-211H cells (Fig. 2). The radiation sensitivity of both cell lines *in vitro* was very similar.

### Radiosensitizing effects of SQAP in a human MPM orthotopically implanted mouse model

At the beginning of the animal experiments, we established orthotopic implanted models of MPM by injecting the human MESO-4 (epithelioid subtype) or MSTO-211H (biphasic subtype) cells into the right thoracic cavities of the mice at 5 × 10^6^ cells/body. All mice were dissected after death, and tumor nodules in the thoracic cavity were observed. The timeline of the study is shown in Figure 3. Because of the high-grade malignancy of MSTO-211H cells, MSTO-211H cell-implanted mice died within 40 days (Fig. 4A). The 50% survival time was 37 days. On the other hand, MESO-4 cell-implanted mice survived for more than 100 days and the 50% survival time was 128 days.

To examine the synergistic effect of SQAP with radiotherapy (RT) in MPM-bearing mice, we examined four groups: control, SQAP (SQAP administration; 2 mg/kg/day, days 0–4), RT (whole body irradiation; 2 or 4 Gy/day, days 0 and 3) and SQAP with RT. We selected MSTO-211H cells because they exhibited a higher malignant potential than the MESO-4 cells. In the model, 2 Gy/day RT (days 0 and 3) prolonged survival when SQAP was administered concurrently (Fig. 4B). Furthermore, 4 Gy/day RT (days 0 and 3) significantly improved the prognoses of MSTO-211H cell-implanted mice when SQAP was concurrently administered (*P* = 0.0143), which suggests that a higher radiation dose was more effective against orthotopically implanted MSTO-211H cells (Fig. 4C).

### Radiosensitizing effects of SQAP in a human MPM ectopically implanted model

**Fig. 5:**
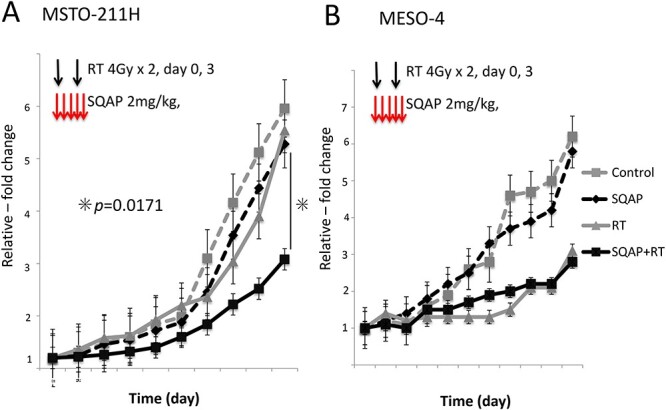
Tumor growth delay assay in ectopic model mice. Relative tumor volume as a function of the days after initiating treatment. Five injections of SQAP (2 mg/kg, i.v.) plus two fractions of irradiation (4 Gy/ fraction) were administered to ectopic MPM model mice implanted with MSTO-211H (control; n = 8, SQAP; n = 8, RT; n = 9, and SQAP combined with RT; n = 7) (A) and MESO-4 (control; n = 4, SQAP; n = 4, RT; n = 4, and SQAP combined with RT; n = 4) (B). Tumors derived from MSTO-211H cells showed greater growth inhibition after combined treatment *in vivo* (A). SQAP, α-sulfoquinovosyl-acylpropanediol; RT, radiation therapy.

To further examine the radiosensitizing effect of SQAP, we established an ectopically implanted mouse model by injecting human MPM cells into the dorsal subcutaneous skin of mice (Fig. 3B). We investigated four groups: control, SQAP (SQAP administration; 2 mg/kg/day, days 0–4), RT (whole body irradiation; 4 Gy/day, days 0 and 3) and SQAP with RT. When the estimated tumor volume reached >200 mm^3^, MPM model mice in each group received the described treatment. In the MSTO-211H cell-implanted model, tumor growth was inhibited significantly in the SQAP with RT group compared with that of the RT group, suggesting an obvious radiosensitizing effect of SQAP only in the MSTO-211H cell-implanted model (*P* < 0.0171; Fig. 5A). In the MESO-4 cell-implanted model, the tumor growth curves of the RT and SQAP with RT groups overlapped (Fig. 5B). The therapies did not harm the general condition of the mice because their body weights were unchanged in all groups (data not shown).

### Cell proliferation and apoptosis in the harvested tumors of ectopic MPM-implanted mice

**Fig. 6:**
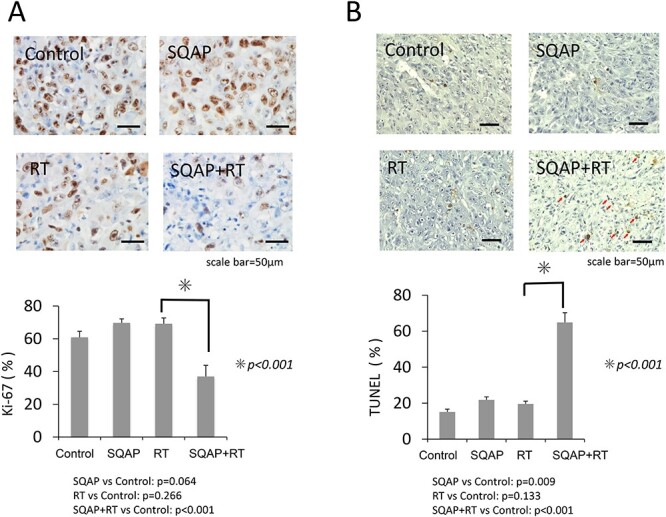
Ki-67 and TUNEL staining of tumor tissues. Ki-67, a cell proliferation marker, is expressed in proliferating cells except during the G0 period and was used as a convalescence prediction factor for the biphasic-type of malignant pleural mesothelioma (MSTO-211H) (control; n = 6, SQAP; n = 8, RT; n = 9, and SQAP combined with RT; n = 7) (A). TUNEL staining is used as an index of apoptosis. SQAP controlled cell proliferation and promoted apoptosis (B). SQAP, α-sulfoquinovosyl-acylpropanediol.

We analyzed the effect of SQAP on MSTO-211H cell proliferation and apoptosis in harvested tumors via immunohistochemistry. Ki-67-positive proliferating cells were decreased significantly in the SQAP with RT group compared with that in the other groups (Fig. 6A).

TUNEL staining showed that positive cells were increased significantly in the SQAP with RT group compared with the other groups (Fig. 6B). The average rates of TUNEL-positive cells were 15.1%, 21.9%, 19.6% and 64.9%, in the control, SQAP, RT and SQAP with RT groups, respectively.

### Evaluation of vascular normalization and reoxygenation of tumors

**Fig. 7:**
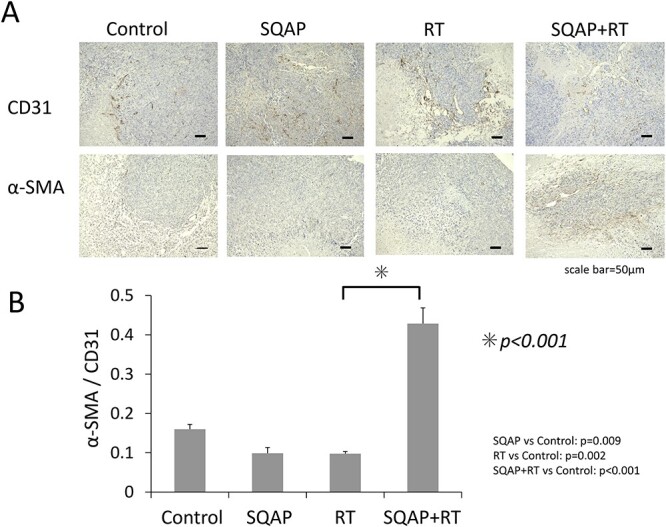
Antiangiogenic effect of SQAP in tumor tissues. Effect of SQAP and/or RT on CD31 and α-SMA in MSTO-211H xenografts. The graph shows quantitative comparisons of CD31 and α-SMA after initiating treatment (control; n = 6, SQAP; n = 8, RT; n = 9, and SQAP combined with RT; n = 7). SQAP induced vascular normalization in the tumor tissue of the combined treatment group. SQAP, sulfoquinovosyl-acylpropanediol; RT, radiation therapy.

Malignant tumors tend to have abnormal vasculature without smooth muscle. Thus, we analyzed α-SMA and CD31 protein expression in the harvested MSTO-211H tumors via immunohistochemistry (Fig. 7) to examine the effects of the therapies. In the control group, many tumor vessels lacked α-SMA protein expression, suggesting an increase in abnormal vessels lacking a smooth muscle layer (Fig. 7A). Conversely, the tumor vessels had regressed in the MSTO-211H tumors of the SQAP with RT group.

The ratio of SMA- to CD31-positive cells was used as a vascular normalization index. The average vascular normalization index ratios were 0.16, 0.10, 0.10 and 0.43, in the control, SQAP, RT and SQAP with RT groups, respectively (Fig. 7B). In the SQAP with RT group, most vessels remaining in the tumors exhibited α-SMA-covered CD31 vessels, which suggests that most vessels had normalized vasculature (*P* < 0.001).

**Fig. 8:**
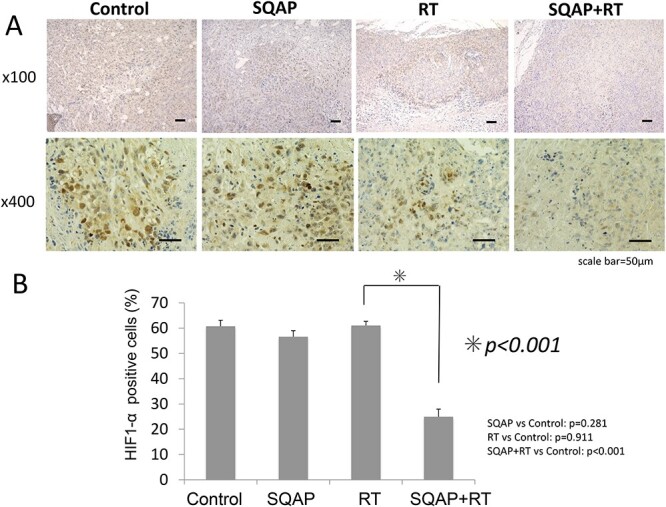
HIF1-α staining of tumor tissue. SQAP reduced HIF-1α protein levels in the tumor tissues (METO-211H) of the combined treatment group (control; n = 6, SQAP; n = 8, RT; n = 9, and SQAP combined with RT; n = 7). HIF-1, hypoxia-inducible factor-1.

HIF1α increases under hypoxic conditions. Immunohistochemical staining using an anti-HIF1α antibody demonstrated that hypoxic cells were significantly reduced in the SQAP with RT group (*P* < 0.001; Fig. 8). Moreover, SQAP reduced the HIF1α protein levels in harvested tumors from the SQAP with RT group. Because HIF1α protein is highly expressed in hypoxic regions, this result indicates that SQAP induced reoxygenation of the tumor tissues.

**Fig. 9:**
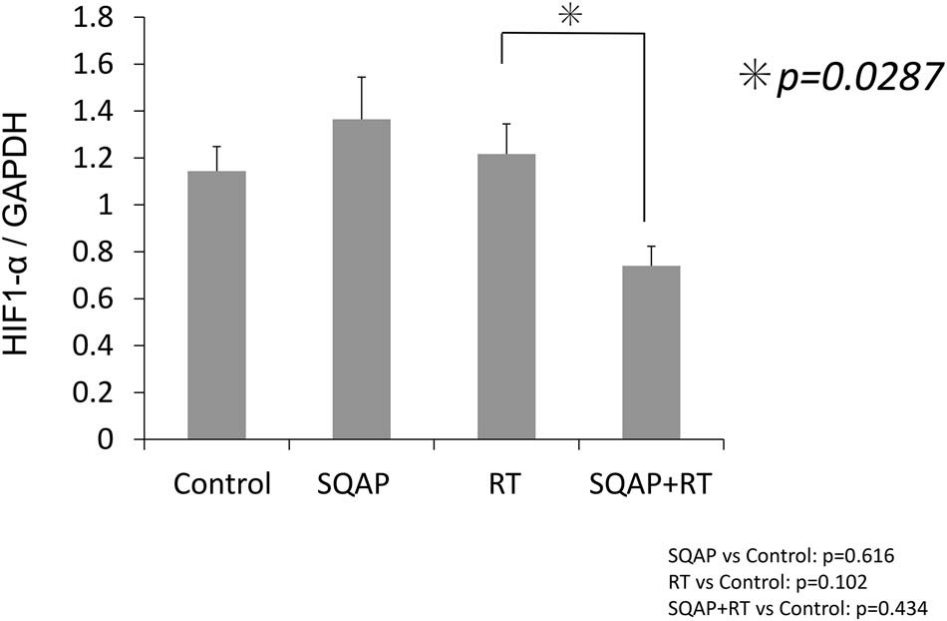
Evaluation of *HIF1* gene expression in tumor tissues. Droplet digital PCR of *HIF1A* in tumors after initiating treatment (control; n = 6, SQAP; n = 7, RT; n = 9, and SQAP combined with RT; n = 7). *HIF1A* expression was downregulated in the tumor tissues of the combined treatment group. HIF-1, hypoxia-inducible factor-1.

We next performed digital droplet PCR to assess differences in *HIF1A* mRNA expression among the groups (Fig. 9). *HIF1α* mRNA was significantly downregulated in the SQAP with RT group (*P* = 0.0287).

## DISCUSSION

RT is an option for cancer therapy; more than 50% of cancer patients will receive RT during their illness and 40% of cured patients received RT as a part of their treatment [22]. In recent years, combinations of RT and other treatment modalities have been developed by focusing on the tumor microenvironment [21–30]. Among these modalities, SQAP has excellent potential for clinical use. SQAP has no harmful effects and administering 800 mg/kg intraabdominally induces no adverse reactions in mice [31]. However, SQAP alone has almost no antitumor effect. SQAG, a derivative of SQAP, functions as a DNA polymerase inhibitor and exhibits antiangiogenic activity *in vitro* at a relatively high dose (30–50 μmol/L) [32,33]. *In vivo*, antiangiogenic activity was observed after intraperitoneally administering 20 mg/kg SQAP [8]. Interestingly however, low doses (1–2 mg/kg) of SQAP delay the tumor growth of solid cancers when combined with radiation in mouse models. The sensitization enhancement ratio of SQAP is 3.0, which is the highest among radiation sensitizers [7,17]. Importantly, tumor pO_2_ elevation by SQAP under RT might not depend on tumor cell type or the host mouse strains [20,34]. The biological effect directly resulted in tumor radiosensitization *in vivo*, and was attributed to hemoglobin allosteric ligand binding causing a release of O_2_ from oxyhemoglobin and increasing tumor pO_2_. Such biological effects have not been previously reported for sulfoglycolipids. Considering the safety, efficacy, and low administration dose, SQAP is superior to previously reported radiation sensitizers for clinical use. Accordingly, SQAP is now used for cancer or thymoma treatment of pet animals under the drug name of Lavurchin^Ⓡ^ [34].

In the present study, SQAP combined with RT suppressed highly malignant MPM tumor growth in animal models, which indicates that SQAP acts as a promising radiation sensitizer even for human MPM. We used two human mesothelioma cell lines. MESO-4 cells originate from epithelioid-type MPM, and MSTO-211H originate from biphasic-type MPM. Clinically, the malignant potential of biphasic-type MPM is worse than that of epithelial-type MPM. Therefore, we considered that MESO-4 and MSTO-211H might have different clinical characteristics and radiosensitivity. Accordingly, *in vitro*, the high radiation dose of 8 Gy induced cell death in both MSTO-211 and MESO-4 cell lines. However, lower radiation doses induced cell death only in MESO-4 cells, which suggests that MESO-4 cells are more sensitive to radiation than MSTO-211H cells. Additionally, MSTO-211H cell lines proliferated more rapidly than MESO-4 cells, which indicates that MSTO-211H cells have a higher malignant potential than MESO-4 cells. In the MESO-4 cell-implanted model *in vivo*, the tumor growth curve of the SQAP with RT group overlapped that of the RT group, possibly because of the high radiation sensitivity of the MESO-4 cells. In the biphasic-type MSTO-211H cell-implanted model, RT or SQAP single treatments did not suppress implanted MSTO-211H tumor growth. However, SQAP with RT suppressed tumor growth, which suggests that SQAP reinforced the RT effect on radiation-resistant MPM. The survival period of the orthotopically implanted MSTO-211H group was shorter than that of the MESO-4 cell-implanted group. Thus, SQAP might have more benefit for RT-tolerating high-grade MPMs such as the biphasic or sarcomatoid types.

Details of the mechanisms of the tumor-suppressing effects of SQAP are unclear. Takakusagi and colleagues reported experimental evidence that demonstrated transient SQAP-induced changes on tumor microenvironment that enhanced tumor oxygenation by facilitating oxygen dissociation from oxyhemoglobin and improving tumor perfusion [20]. Murine squamous cell carcinoma of SCCVII and human lung cancer of A549 tumors were grown by injecting tumor cells into the hind legs of mice. Five days of daily radiation (2 Gy) combined with intravenous injection of SQAP (2 mg/kg) 30 minutes prior to irradiation significantly delayed growth of tumor xenografts. Three days of daily treatment improved tumor oxygenation and decreased tumor microvascular density on T2^*^-weighted images with USPIO, suggesting vascular normalization. Therefore, SQAP-mediated sensitization to radiation *in vivo* can be attributed to increased tumor oxygenation. Recent *in vivo* studies revealed that SQAP induced the degradation of HIF-1α and then decreased the expression of histone deacetylase (HDAC) [35], which is related to the abnormal proliferation of cancer cells [36,37].

Based on these previous reports and this study, we presume that downregulation of HIF1-α expression might be important for the antitumor effect in this *in vivo* model because HIF1α is believed to be a critical factor for radiation sensitivity in tumors. Because HIF1α regulates tumor angiogenesis, suppressing HIF1α normalizes the vasculature by decreasing the abnormal vasculature generation in tumors. Subsequently, normalized vessels supply oxygen, which reoxygenates tumors. Irradiation in this state promotes apoptosis and suppresses malignant cell proliferation. Accordingly, the combined therapy in MSTO-211H tumors significantly reduced the Ki-67 positivity rate and significantly increased the TUNEL positivity rate after 50 days. HIF1α is believed to exert antiangiogenic effects and is related to tumor vessel abnormalities. Hence, HIF-1α mRNA and protein expression levels were decreased significantly in the SQAP with RT group compared with those of the other groups. Accordingly, CD31 and SMA immunostaining significantly normalized the vasculature in the SQAP with RT group.

A limitation of the present study was that we did not analyze focal adhesion kinase (FAK). Izaguirre-Cabonell and colleagues previously identified five SQAP-binding proteins: sterol carrier protein 2, multifunctional enzyme type 2, proteasomal ubiquitin receptor, UV excision repair protein and FAK. Among the binding proteins, analyzing the inhibition of FAK phosphorylation might be important because FAK phosphorylation is the mechanism for the antiangiogenic activity of SQAP [15]. Because HIF1 and FAK are closely related, elucidating the signal cascade enhancement from FAK to HIF1 might be important for suppressing MPM growth. In our future research, we aim to identify the ideal timing and dose for both SQAP administration and irradiation for clinical application.

In conclusion, SQAP with RT suppressed MPM tumor growth in mouse models, especially in radiation-resistant biphasic-type MPM. Downregulating the HIF1α cascade with vascular normalization might be a mechanism for this effect in MPM tumors. We believe that SQAP may be a novel radiosensitizer for treating MPM and should be applied in clinical trials.

## PRESENTATION AT A CONFERENCE

American Thoracic Society 2018, San Diego.
